# A randomized clinical trial to determine the efficacy of manufacturers’ recommended doses of omega-3 fatty acids from different sources in facilitating cardiovascular disease risk reduction

**DOI:** 10.1186/1476-511X-13-99

**Published:** 2014-06-21

**Authors:** Maggie Laidlaw, Carla A Cockerline, William J Rowe

**Affiliations:** 1 Nutrasource Diagnostic Inc., 120 Research Lane, University of Guelph Research Park, Suite 203, Guelph N1G0B4, Ontario, Canada

**Keywords:** Omega-3 supplements, Cardiovascular disease, Risk biomarkers

## Abstract

**Background:**

Omega-3 fatty acids confer beneficial health effects, but North Americans are lacking in their dietary omega-3-rich intake. Supplementation is an alternative to consumption of fish; however, not all omega-3 products are created equal. The trial objective was to compare the increases in blood levels of omega-3 fatty acids after consumption of four different omega-3 supplements, and to assess potential changes in cardiovascular disease risk following supplementation.

**Methods:**

This was an open-label, randomized, cross-over study involving thirty-five healthy subjects. Supplements and daily doses (as recommended on product labels) were:

Concentrated Triglyceride (rTG) fish oil: EPA of 650 mg, DHA of 450 mg

Ethyl Ester (EE) fish oil: EPA of 756 mg, DHA of 228 mg

Phospholipid (PL) krill oil: EPA of 150 mg, DHA of 90 mg

Triglyceride (TG) salmon oil: EPA of 180 mg, DHA of 220 mg.

Subjects were randomly assigned to consume one of four products, in random order, for a 28-day period, followed by a 4-week washout period. Subsequent testing of the remaining three products, followed by 4-week washout periods, continued until each subject had consumed each of the products. Blood samples before and after supplementation were quantified for fatty acid analysis using gas chromatography, and statistically analysed using ANOVA for repeated measures.

**Results:**

At the prescribed dosage, the statistical ranking of the four products in terms of increase in whole blood omega-3 fatty acid levels was concentrated rTG fish oil > EE fish oil > triglyceride TG salmon oil > PL krill oil. Whole blood EPA percentage increase in subjects consuming concentrated rTG fish oil was more than four times that of krill and salmon oil. Risk reduction in several elements of cardiovascular disease was achieved to a greater extent by the concentrated rTG fish oil than by any other supplement. Krill oil and (unconcentrated) triglyceride oil were relatively unsuccessful in this aspect of the study.

**Conclusion:**

For the general population, the form and dose of omega-3 supplements may be immaterial. However, given these results, the form and dose may be important for those interested in reducing their risk of cardiovascular disease.

**Trial registration:**

ClinicalTrials.gov: NCT01960660.

## Background

The health benefits of omega-3 fatty acids (ω-3 FAs) have been extensively examined in many published studies, and these benefits have been observed in patients with a diversity of conditions and diseases, including cardiovascular disease (e.g. atrial fibrillation, atherosclerosis, thrombosis, inflammation, sudden cardiac death, etc.), age-related cognitive decline, periodontal disease, rheumatoid arthritis, etc. [1-9]. Although the consumption of fatty fish is the recommended mode of incorporation of ω-3 FAs into the North American diet, fish consumption in North America is much lower than in parts of European and Asian countries [10-19].

Supplementation with ω-3 FAs may provide a viable alternative to fish consumption. In a recent U.S. survey, sixty-eight percent of adults take some form of supplementation, and of those, nineteen percent consume ω-3 supplements [20]. Not all ω-3 supplements are created equal, however, and there is conflicting information in the scientific literature with regard to the relative bioavailability of different ω-3 supplements. The most important ω-3 FAs, eicosapentaenioc acid (EPA) and docosahexaenoic acid (DHA) may be present in an ethyl ester (EE) form, in a triglyceride (TG) form, as Free Fatty Acid (FFA) or as phospholipids (PL). It has been suggested that the form in which the ω-3FA is consumed may affect its relative bioavailability. For example, if the ω-3FA is in the *sn*-2 position on the TG glycerol backbone, then after cleavage of the long-chain fatty acids (LCFAs) on the *sn-1* and *sn-3* positions, the *sn-2* FA, still attached to the glycerol backbone, is preferentially absorbed as a monoglyceride by passive diffusion, while the cleaved LCFAs require a protein mediator for absorption. Additionally, the distribution of these long-chain fatty acids (LCFAs) between the inner and outer positions of intestinally-resynthesized TGs may influence their incorporation into plasma lipoprotein fractions [21].

The superior bioavailability of TG versus EE forms of ω-3FA was first identified by several researchers more than thirty years ago [22-24]. This finding has been confirmed more recently, although there have been some contrary findings [25-29]. Research on the relative bioavailability of PL forms of ω-3FA is limited and inconclusive. In the most recent publication on this issue, krill oil, the most common phospholipid supplement, was not significantly different than re-esterified TG or EE in bioavailability of DHA or EPA + DHA, although there was a trend towards an increased bioavailability of EPA with krill oil [30]. However, the krill oil used in this study contained significant amounts of both PL and FFAs, and the latter has been shown to exhibit slightly superior bioavailability of ω-3FA, compared to the EE form [31].

Omega-3 supplement manufacturers appear to be making use of published bioavailability literature when they recommend suitable dosages on the labels of their products. Thus, there is a wide range of recommended dosages available, such that the consumer may not be achieving an intake or blood level of ω-3 FAs that is conducive to possible health improvements and/or disease risk reduction. The objective of this study was to compare the increases in blood levels of omega-3 fatty acids after consumption of four different omega-3 supplements, and to assess potential changes in cardiovascular disease (CVD)risk following supplementation.

The daily dose of each supplement, as recommended on the label by the manufacturer, was as follows:

Concentrated Triglyceride (rTG) fish oil: EPA of 650 mg, DHA of 450 mg

Ethyl Ester (EE) fish oil: EPA of 756 mg, DHA of 228 mg

Phospholipid (PL) krill oil: EPA of 150 mg, DHA of 90 mg

Triglyceride (TG) salmon oil: EPA of 180 mg, DHA of 220 mg.

All samples had tocopherols added for antioxidant function, although none listed the quantity on product labels. The PL and TG supplements also contained the naturally-occurring antioxidant astaxanthin, at 1.5 mg and 2.5 μg respectively. Each supplement was analysed for omega-3 fatty acid content at the beginning and end of the trial. Levels of omega-3 fatty acids were unchanged, and matched target values.

## Results

A total of 46 prospective participants were pre-screened, of whom 35 were found to be eligible. They were enrolled, randomized and received at least one dose of study supplement. Three participants prematurely discontinued, two of whom experienced minor adverse events and one whose time became limited. Data from 32 participants was available for analysis.

### Subject characteristics

Baseline subject characteristics are presented in Table 1. There were no significant changes in any of these parameters during the trial, nor were there any serious adverse events. Mean compliance rate was 99%, and no participant consumed less than 80% of their study medication.

**Table 1:** Baseline characteristics of trial participants, by gender

	**Group**	**N**	**AGE**	**Gender**	**HT**^**1**^	**WT**^**2**^	**BPS**^**3**^	**BPD**^**4**^	**BMI**
**Mean**	All	35	35	ALL	1.726	77.08	120.7	78.8	25.81
**SD**^**5**^			14		0.081	20.09	13.2	10.2	6.13
**Mean**	Males	18	29	Male	1.764	80.89	126.2	77.9	26.04
**SD**			14		0.045	10.90	13.3	11.9	3.69
**Mean**	Females	17	40	Female	1.685	73.04	114.9	79.7	25.57
**SD**			13		0.093	26.42	10.6	8.3	8.08

^1^HT: Height (m); ^2^WT: Weight (kg): ^3^BPS: Systolic blood pressure; ^4^BPD: Diastolic Blood.

Pressure; ^5^SD: Standard Deviation.

### Fatty acid results

In statistical testing for effect of order of treatment, there were no significant difference among the four orders of treatment, and baseline levels of all biomarkers of interest were not significantly different from one another when comparing the four groups (data not shown).

Abbreviations used in the following tables, and not yet defined, are as follows:

AA: Arachidonic Acid; DPA: Docosapentaenoic Acid; ω-3 FA: omega-3 fatty acids; ω-6-FA: omega-6 fatty acids; ω3:ω6: omega-3:omega-6 Ratio; ω-6: ω:3: omega-6:omega-3 Ratio; CP: Comparator product; SD: Standard Deviation.

**Figure 1:**
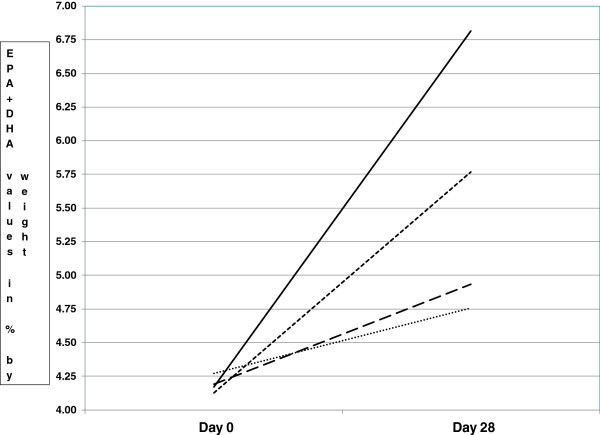
Mean levels of EPA + DHA for four supplement groups at Day 0 and Day 28.

Fatty acids and calculated fatty acid combination results are presented in Table 2.Figure 1 illustrates the EPA + DHA changes during the supplementation period, for the four comparator products.

**Table 2:** Values for selected fatty acids and fatty acid calculated ratios* on Day 0 and Day 28, by treatment

**Treatment**	**Visit**	**AA**	**EPA**	**DPA**	**DHA**	**EPA + DHA**	**AA:EPA**	**EPA + DPA + DHA**	**ω-3 FA**	**ω-6 FA**	**ω-3:ω-6**	**ω-6:ω-3**
rTG	Day 0	12.217 ± 2.091	1.175 ± 0.438	1.344 ± 0.303	2.997 ± 0.672	4.172 ± 0.923	11.886 ± 4.901	5.516 ± 0.983	6.333 ± 1.063	43.156 ± 3.999	0.148 ± 0.029	7.022 ± 1.484
	Day 28	11.666 ± 1.854	2.644 ± 0.717	1.595 ± 0.290	4.174 ± 0.657	6.818 ± 1.190	4.738 ± 1.523	8.418 ± 1.165	9.140 ± 1.239	40.530 ± 4.168	0.228 ± 0.043	4.516 ± 0.771
EE	Day 0	11.550 ± 2.284	1.065 ± 0.367	1.356 ± 0.287	3.063 ± 0.844	4.128 ± 1.004	12.164 ± 4.938	5.487 ± 1.168	6.242 ± 1.193	41.911 ± 4.634	0.151 ± 0.034	6.991 ± 1.674
	Day 28	11.235 ± 2.129	2.449 ± 0.858	1.544 ± 0.298	3.318 ± 0.763	5.767 ± 1.249	5.058 ± 1.783	7.311 ± 1.311	8.067 ± 1.289	40.339 ± 4.102	0.201 ± 0.033	5.103 ± 0.853
PL	Day 0	11.57 ± 2.134	1.147 ± 0.463	1.352 ± 0.291	3.123 ± 0.759	4.269 ± 0.968	11.576 ± 5.007	5.628 ± 1.088	6.428 ± 1.203	42.300 ± 5.157	0.154 ± 0.036	6.784 ± 1.341
	Day 28	11.316 ± 1.897	1.440 ± 0.487	1.502 ± 0.269	3.317 ± 0.728	4.756 ± 0.942	8.836 ± 3.449	6.258 ± 1.045	6.951 ± 1.075	40.849 ± 4.168	0.171 ± 0.030	6.002 ± 11.04
TG	Day 0	11.698 ± 2.667	1.131 ± 0.426	1.446 ± 0.354	3.061 ± 0.763	4.192 ± 0.942	11.568 ± 4.508	5.638 ± 1.098	6.396 ± 1.137	41.791 ± 5.499	0.155 ± 0.029	6.687 ± 1.177
	Day 28	11.345 ± 1.826	1.425 ± 0.465	1.460 ± 0.291	3.508 ± 0.640	4.932 ± 0.865	8.963 ± 4.037	6.388 ± 0.850	7.148 ± 1.007	41.088 ± 3.135	0.175 ± 0.032	5.872 ± 1.002

*All values in % by weight, Mean ± Standard Deviation.

The changes and percentage changes between Day 0 and Day 28 for the fatty acids of interest, as well as the statistical significance, are shown in Table 3.

**Table 3:** Change and percentage change (in% by weight) in selected fatty acids and fatty acid ratios of interest between Day 0 and Day 28, by treatment, with statistical significance

**Treatment**	**AA**		**EPA**		**DPA**		**DHA**		**EPA + DHA**		**AA:EPA**		**EPA + DPA + DHA**		**ω -3 FA**		**ω -6 FA**		**ω-3:ω-6 Ratio**		**ω-6:ω-3 Ratio**	
	**Δ**	**% Δ**	**Δ**	**% Δ**	**Δ**	**% Δ**	**Δ**	**% Δ**	**Δ**	**% Δ**	**Δ**	**% Δ**	**Δ**	**% Δ**	**Δ**	**% Δ**	**Δ**	**% Δ**	**Δ**	**% Δ**	**Δ**	**% Δ**
rTG mean	-0.551	-3.27	1.469	151.1***	0.251	22.8**	1.177	44.6***	2.646	69.8***	-7.148	-56.7***	2.903	56.9***	2.807	47.5***	-2.626	-5.78**	0.080	57.6***	-2.507	-34.4***
rTG SD	1.770	14.60	0.691	102.69	0.312	28.01	0.628	36.49	1.150	44.37	4.271	13.62	1.218	34.63	1.244	28.671	3.889	8.970	0.033	30.403	1.342	11.366
EE mean	-0.316	-0.98	1.384	155.0***	0.189	17.0*	0.255	12.90	1.638	47.5***	-7.107	-54.3***	1.824	39.4***	1.826	33.9***	-1.572	-3.200	0.050	37.4***	-1.888	-24.7***
EE SD	2.207	19.54	0.847	123.65	0.273	24.74	0.656	31.80	1.365	49.05	4.745	18.13	1.521	41.46	1.497	34.923	4.153	9.537	0.029	28.093	1.413	13.871
PL mean	-0.260	-0.73	0.293	34.9*	0.150	14.5*	0.194	8.93	0.487	14.5*	-2.740	-17.2*	0.637	13.5***	0.523	10.200	-1.450	-2.720	0.017	13.2*	-0.782	-10.2*
PL SD	1.876	16.32	0.528	45.48	0.290	24.65	0.512	20.08	0.874	23.43	4.109	34.78	0.974	19.14	1.131	18.698	4.559	10.214	0.024	16.039	0.921	12.839
TG mean	-0.353	0.24	0.294	35.7**	0.014	4.37	0.447	18.9*	0.740	21.3**	-2.606	-17.9*	0.751	16.6***	0.752	14.2**	-0.703	-0.628	0.020	13.4*	-0.815	-11.4**
TG SD	2.206	20.22	0.493	43.34	0.301	21.60	0.596	27.34	0.866	25.65	4.249	34.61	1.118	23.20	1.161	20.229	4.509	10.030	0.020	12.827	0.791	11.331

∆ = change; %∆ = percent change; SD = Standard deviation; ***p-value - <0.0001 - <0.001; **p-value 0.001 – <0.01; ***p-value 0.01 – < 0.05.

**Figure 2:**
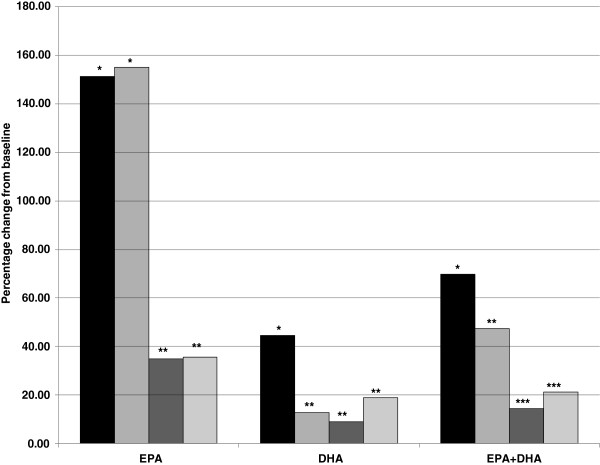
Mean percentage change in EPA, DHA and EPA + DHA levels from Day 0 to Day 28, with statistical comparisons among the four comparator products.

From Table 3, it is noted that the increase in omega-3 fatty acids after rTG supplementation is statistically greater than for the other products. Also, the AA:EPA ratio is statistically lower after rTG supplementation than for the other products.Figure 2 illustrates the EPA, DHA and EPA + DHA percentage changes and the results of statistical comparisons of those changes, among the comparator products.

As with Figure 1, Figure 2 demonstrates that rTG supplementation produced a significant improvement over the other three products in both DHA increase and EPA + DHA increase, from Day 0 to Day 28. Supplementation with this product also produced a statistically higher increase in whole blood EPA than the PL and TG products. From Tables 2 and 3, and Figures 1 and 2, it appears that the order of efficacy in raising the blood levels of the ω-3FA of interest (primarily EPA and DHA) is as follows: rTG > EE > TG > PL, based on the percentage changes and relative p-values for the various ω-3FAs and FA combination and ratio increases, as well as the statistical comparisons among the four comparator products. These results are to be expected, given the relative daily doses of EPA and DHA in each of the supplements. These were the daily doses recommended by the manufacturers, as listed on each label for the four supplements.

Comparisons are more appropriate for supplements with similar intakes of specific fatty acids. For example, the rTG and EE groups had somewhat similar daily intakes of EPA, 650 mg and 756 mg, respectively. In Table 3, it is noted that the mean percentage increase in whole blood EPA for the rTG and EE groups was very similar, at 151.1% and 155.0%, respectively. For DHA, the intake for the rTG group was twice that of the EE group; however, the whole blood percentage increase in DHA was 44.6% for the rTG group but only 12.9% for EE, an almost fourfold difference. The PL group intake of EPA was similar to that of the TG group (150 and 180 mg/day, respectively) and the mean whole blood EPA percentage increases were almost identical, at 34.9% and 35.7%, respectively. Finally, the DHA intake of the EE and TG groups were only 3.6% apart, at 228 and 220 mg/day, respectively, yet the mean whole blood percentage changes were 12.9% and 18.9% respectively, an almost 50% difference.

The data was also analysed for gender differences, and some modest differences were noted. For example, the EPA% change between the EE and the TG supplements was statistically significant for females, but not quite significant for males (p-value of <0.0001 and 0.0693, respectively). Similarly, both the DHA change and% change, when comparing the rTG and TG supplements, were significantly different for females (p-value of 0.0002 and 0.0182, respectively), whereas for males these same variables were not significantly different (p-value of 0.0736 and 0.2753, respectively). Otherwise, in pair-wise comparisons, statistically significant changes were similar in males and females, for EPA and DHA.

### Extrapolation of data for a 1,000 mg intake

Given the differences in intake levels for the four supplements, an attempt was made to extrapolate the data for an intake of 1,000 mg/day of each omega-3 fatty acid. For three of the four supplements in this trial, some subjects actually experienced negative results from Day 0 to Day 28, i.e. the level of one or more of their long-chain omega-3 fatty acids actually decreased. For example, although none of the subjects consuming the rTG and the EE products experienced a decrease in EPA levels from Day 0 to Day 28, eight of the subjects in the krill oil PL group, and six consuming the TG supplement did experience a drop in EPA levels. Similarly, the blood DHA levels for a number of subjects in the EE, PL and TG groups decreased from Day 0 to Day 28 (11, 13 and 7 subjects, respectively). All of the capsule counts for these subjects indicated full or close-to-full compliance; however, capsule count is not always the best indicator of compliance. In this trial, using % change in blood EPA levels (often a more accurate measure of compliance) was not appropriate because of the great variation in the actual daily intake dosage of EPA (ranging from a low of 150 mg/day to a high of 756 mg/day). Thus, results were taken at face value, assuming full compliance.

Because an extrapolation calculation involving a negative result (i.e. decrease in the level of an omega-3 biomarker from Day 0 to Day 28) will merely increase its “negativity”, negative results were dealt with in two different ways: removal of negative values entirely, or allocating all negative values at 0. Table 4 illustrates these extrapolated results.

**Table 4:** Extrapolated results for an intake of 1,000 mg/day of EPA or DHA

**All data included**		**EPA**		**DHA**	
**TRT**	**Statistic**	**%∆**	**Extrapolated % ∆**	**%∆**	**Extrapolated % ∆**
**rTG**	**Mean**	151	232	45	99
	**SD**	103	158	36	81
**EE**	**Mean**	155	205	13	57
	**SD**	124	164	32	139
**PL**	**Mean**	35	232	9	99
	**SD**	45	303	20	223
**TG**	**Mean**	36	198	19	86
	**SD**	43	241	27	124
Negative data deleted*		**EPA**		**DHA**	
**TRT**	**Statistic**	**%∆**	**Extrapolated % ∆**	**%∆**	**Extrapolated % ∆**
**rTG**	**Mean**	151	232	45	99
	**SD**	103	158	36	81
**EE**	**Mean**	155	205	26	113
	**SD**	124	164	32	141
**PL**	**Mean**	53	354	22	240
	**SD**	36	242	16	180
**TG**	**Mean**	50	278	27	123
	**SD**	33	185	25	115
Negative data allocated 0 value**		**EPA**		**DHA**	
**TRT**	**Statistic**	**%∆**	**Extrapolated % ∆**	**%∆**	**Extrapolated % ∆**
**rTG**	**Mean**	151	232	45	99
	**SD**	103	158	36	81
**EE**	**Mean**	155	205	17	74
	**SD**	124	164	29	125
**PL**	**Mean**	40	266	13	142
	**SD**	39	260	16	182
**TG**	**Mean**	41	226	21	96
	**SD**	36	200	25	113

*Any subject data in which the EPA or DHA values decreased from Day 0 to Day 28 were deleted from the extrapolation calculations for the appropriate omega-3 fatty acid.

**Any subject data in which the EPA and/or DHA values decreased from Day 0 to Day 28 were given a value of zero (0) for the omega-3 fatty acid(s) in question.

### Potential for cardiovascular disease risk reduction

One of the biomarkers employed to assess the potential ω-3 FA effect on CVD disease risk reduction was the whole blood OmegaScore™. This test is a combined measure of EPA + DPA + DHA, and high vs. low quartiles are linked to low or high risk of sudden death [32]. From this biomarker, correlation equations have been formulated to provide three other biomarker scores, the Omega-3 Serum Equivalence Score (ω-3 SES), from which heart disease risk reduction may be extrapolated [33], the EPA + DHA Serum Equivalence Score (E + DSES), from which risk of death from fatal ischemic heart disease may be extrapolated [34], and the Omega-3 Red Blood Cell Equivalence Score (ω-3RBCES), also known as the Omega-3 Index, from which protection against sudden myocardial infarction may be estimated [35].

Using the directly measured OmegaScore^®^ results, the three associated scores were calculated for each subject, and the risk level for each subject was given a risk ranking based on the cut-off points for each of the four parameters listed previously. These cut-off points are presented in Table 5. Each subject’s risk ranking change from Day 0 to Day 28 was rated as Good, Neutral or Poor, based on the following:

Good: Subject’s risk ranking changed from a higher risk to a lower risk, e.g. high risk to moderate risk, or moderate risk to low risk, etc.

Neutral: Subject’s initial risk ranking of low or moderate risk did not change from Day 0 to Day 28.

Poor: Subject’s risk ranking changed from a lower risk to a higher risk, or, in the case of an initial high or very high risk, did not change from Day 0 to Day 28.

**Table 5:** Cut-off points for four biomarkers associated with cardiovascular risk

**Biomarker**	**Risk condition**	**Very high risk**	**High risk**	**Moderate risk**	**Low risk**
**OmegaScore™ (EPA + DPA + DHA)**	**Sudden death**	<4.3	4.3-5.1	5.1-6.1	>6.1
**ω-3 Serum Equivalence Score**	**Heart disease**	N/A	<5.1	5.1-7.2	>7.2
**EPA + DHA Serum Equivalence Score**	**Fatal ischemic heart disease**	N/A	<3.5	3.5-4.5	>4.5
**ω-3 Red Blood Cell Equivalence Score**	**Myocardial infarction**	N/A	<5.2	5.2-8.0	>8.0

Table 6 illustrates the assessment of the four supplements, vis-à-vis these ratings. From Tables 5 and 6, it should be noted that the EPA + DHA Serum Equivalence Score cut-off point from moderate to low risk is relatively lower than for the other calculated biomarkers, with the result that many of the subjects’ initial EPA + DHA Serum Equivalence Scores were already higher than the low risk cut-off point. This meant that the potential for positive change in this biomarker was limited; therefore, the total scores were calculated with and without this biomarker included, and they are also presented in Table 6. At the daily doses directed by the manufacturer, the concentrated triglyceride (rTG) supplement was most successful in reducing risk across the parameters of OmegaScore™, Omega-3 Serum Equivalence Score and the Omega-3 Red Blood Cell Equivalence Score, with the ethyl ester supplement being quite similar, and the krill and non-concentrated triglyceride supplements being less successful.

**Table 6:** Number of subjects who rated as Good, Neutral or Poor in terms of CVD risk reduction, from Day 0 to Day 28

**Type of FA**	**Score**	**OS™(EPA + DPA + DHA)**	**ω-3 SES**	**E + DSES**	**ω-3 RBCES**	**Total**	**Total minus E + DSES**
**rTG**	**Good**	26	26	9	27	**88**	**79**
**Concentrated**	**Neutral**	6	6	23	5	**40**	**17**
**Triglyceride**	**Poor**	0	0	0	0	**0**	**0**
**EE**	**Good**	19	23	10	16	**68**	**58**
**Ethyl ester**	**Neutral**	12	6	21	15	**54**	**33**
	**Poor**	1	3	1	1	**6**	**5**
**PL**	**Good**	14	12	7	10	**43**	**36**
**Phospho-**	**Neutral**	15	14	23	19	**71**	**48**
**lipid (krill)**	**Poor**	3	6	2	3	**14**	**12**
**TG**	**Good**	18	12	7	9	**46**	**39**
**Triglyceride**	**Neutral**	10	15	25	21	**71**	**46**
	**Poor**	4	5	0	2	**11**	**11**

OS™: OmegaScore™; ω-3 SES: Omega-3 Serum Equivalence Score; E + DSES: EPA + DHA Serum Equivalence Score; ω-3 RBCES: Omega-3 Red Blood Cell Equivalence Score (Omega-3 Index).

As noted previously, a “neutral” rating was given to subjects whose risk category at Day 0 was moderate or low, and whose risk category had not changed by Day 28. However, it could be argued that for those subjects who were initially categorised as low-risk, no change by Day 28 could be considered “good” rather than “neutral”, given that there is no better category than low-risk, and therefore no improvement in that category is possible. In addition, these subjects did not change from the low-risk category to a higher-risk one. Table 7 is a representation of the same calculations as before, except that subjects who were categorised as low-risk initially, and who remained low-risk at Day 28, were given a “good” rating than a “neutral” rating. This readjustment did not alter the overall rankings of the four supplements, although, as expected, it did increase the number of “good” responses for each supplement.

**Table 7:** Adjusted* number of subjects who scored Good, Neutral or Poor in terms of CVD risk reduction, from Day 0 to Day 28

**Type of FA**	**Score**	**OS™(EPA + DPA + DHA)**	**ω-3 SES**	**E + DSES**	**ω-3 RBCES**	**Total**	**Total minus E + DSES**
**rTG**	**Good**	32	30	32	27	**121**	**89**
**Concentrated**	**Neutral**	0	2	0	5	**7**	**7**
**Triglyceride**	**Poor**	0	0	0	0	**0**	**0**
**EE**	**Good**	30	26	31	16	**103**	**72**
**Ethyl ester**	**Neutral**	0	3	0	15	**18**	**18**
	**Poor**	2	3	1	1	**7**	**6**
**PL**	**Good**	24	15	30	10	**79**	**49**
**Phospho-**	**Neutral**	5	11	0	19	**35**	**35**
**lipid (krill)**	**Poor**	3	6	2	3	**14**	**12**
**TG**	**Good**	26	17	30	9	**82**	**52**
**Triglyceride**	**Neutral**	2	10	0	21	**33**	**33**
	**Poor**	4	5	2	2	**13**	**11**

OS™: OmegaScore™; ω-3 SES: Omega-3 Serum Equivalence Score; E + DSES: EPA + DHA Serum Equivalence Score; ω-3 RBCES: Omega-3 Red Blood Cell Equivalence Score.

*“neutral” numbers have been adjusted to exclude subjects whose initial ranking for any of the biomarkers was low risk, and whose ranking did not change from Day 0 to Day 28. These subjects have been re-rated as “good”.

## Discussion

From the results, it has clearly been shown that the concentrated rTG fish oil supplement, at the prescribed daily dose, produces a greater increase in whole blood ω-3FAs than the EE fish oil supplement, the krill oil PL supplement and the salmon oil TG supplement, at their prescribed doses. Both the EPA + DHA combination and DHA alone exhibited the greatest increase with the rTG supplement, and the greatest decrease in ω-6 FAs. For the increase in EPA alone, the concentrated TG fish oil supplement is similar to the EE fish oil supplement in this regard, but much higher that the krill oil PL and salmon oil TG supplements. The percentage change in EPA for the concentrated rTG fish oil and EE fish oil supplement is more than four times that of the krill oil and salmon oil supplements.

For DHA, this increase in the concentrated rTG fish oil supplement is illustrated effectively in Table 3, where the percentage increase in DHA for the concentrated rTG fish oil product is 44%, which is highly significant and much greater than for any of the other supplements. Only the other TG supplement, derived from salmon oil , showed any significant increase, but at a much lower level of significance than the concentrated rTG fish oil supplement, i.e. P = 0.0137 vs. p < 0.0001, respectively (data not shown). In order of best to worst, in terms of significant beneficial changes in ω-3FA biomarkers, in combinations and in calculated ratios, the ranking is concentrated rTG fish oil > EE fish oil > Salmon oil TG > Krill Oil PL. Both the krill oil and the salmon oil TG produced a smaller increase in blood levels of most of the ω-3FA biomarkers of interest, when compared to concentrated rTG fish oil and EE fish oil.

However, given the considerable variability in the supplement doses given to trial subjects, all of the above is to be expected, i.e. the higher the dose, the greater the rise in fatty acids of interest. In order to more accurately compare the four supplements, the results were extrapolated for an intake of 1,000 mg, but this was less than successful, given the considerable number of subjects in some of the groups whose blood levels of some omega-3 fatty acids decreased from Day 0 to Day 28. For example, the krill oil EPA extrapolated increase was 232% with all subjects’ data included, 354% with all negative values deleted, and 266% with all negative values replaced with a zero. However, neither of these latter two values is any more accurate that the value with all data included, as the fact remains that in 8 of 35 subjects in the PL group, blood levels of EPA actually decreased in value during the supplementation period. Because of the difficulties encountered in data extrapolation, i.e. the inaccuracies inherent in the methodology, given the negative results for some subjects, a statistical analysis of this data was not considered to be warranted.

Given the high supplement levels of EPA in the rTG and EE groups, relative to the PL and TG groups, (650 and 756 vs. 150 and 180 mg/day, respectively) one could assume that these groups’ blood EPA values should have increased, and this was the case; indeed, none of these subjects reduced EPA levels, and this tends to reinforce the belief of good compliance with the trial protocol. That being the case, there is no reason to believe that these same subjects were any less compliant when consuming the other two products in this crossover trial, particularly when subjects consumed the four products in different and random order, i.e. they were not suffering from “intake fatigue” towards the end of the study, given that at least some of them consumed the latter two products (PL and TG) at the beginning or middle of the study. Thus, one may conclude that the negative values obtained for the EE, PL and TG groups are an accurate reflection of what transpired in the trial.

One explanation of the negative results for some of the subjects in the latter two groups (PL and TG) is the possibility that these subjects were consumers of a significant amount of fish prior to the study, and that the amount of EPA and DHA in the supplements for these groups was insufficient to match their previous intake from food, prior to supplementation. In future designs, it would be useful to ask subjects to refrain from consuming any fish products for an appropriate amount of time prior to study onset, and to supplement all subjects with a nominal amount of EPA and DHA for a run-in period prior to the commencement of the trial.

Although only small differences in gender comparisons were noted, the number of subjects in each group was small, i.e. 18 males and 17 females, and this may have been inadequate for accurate statistical comparison, such that it was insufficiently powered to detect gender differences. In order to more fully examine possible gender differences, future recruitment of a larger number of subjects in each group would permit more in-depth analysis of possible gender differences following omega-3 supplementation.

Of greater interest to the general population, and particularly to the significant number of North Americans at risk for cardiovascular disease, are the effects of supplementation on CVD risk reduction. The results for the measured and derived biomarkers specifically related to aspects of cardiovascular disease risk reduction clearly illustrate that the consumer must give careful consideration to the type and dosage of supplement they choose. For example, fewer than half of the subjects who consumed the krill oil supplement, at the recommended dosage, reduced the risk for sudden death, heart disease, death from fatal ischemic heart disease and sudden myocardial infarction, according to the measured and calculated indices. The majority experienced no benefit, and a few actually experienced a possible increase in risk.

Conversely, the vast majority of subjects who consumed the concentrated rTG fish oil were projected to reduce their risk of the aforementioned CVD-related conditions, except for the risk of death from fatal ischemic heart disease, where most of the subjects consuming each of the supplements experienced no change in this risk. Results for the EE fish oil group were somewhat better than for the krill oil PL or TG fish oil group, but not as good as those of the concentrated fish oil rTG group.

The fatty acid analyses employed in this study did not include the isolation of serum or plasma phospholipids, or of red blood cell phospholipids. These analyses would more fully reflect the concentrations of ω-3 FAs available for further metabolism. It is at this level of metabolism that ω-3 FAs exhibit their greatest beneficial effects; for example, as anti-inflammatory omega-3 prostaglandin precursors, they compete metabolically with pro-inflammatory ω-6 precursors [36-38]. In any future study, it would be useful to examine the concentrations of ω-3 FAs in membrane phospholipids, vis-à-vis serum phospholipid fatty acid analyses. Also, levels of lipid peroxides in supplements and in subjects’ plasma samples would be useful as an indicator of any deterioration of long chain fatty acids in the supplements.

In addition, a head-to-head comparison of the supplements utilised in this trial, at equivalent doses of EPA and DHA, would be useful in determining their relative bioavailability and their efficacy in increasing blood levels of omega-3 fatty acids, and in reducing CVD risk. In many omega-3 clinical trials, compliance is measured both by pill count before and after supplementation, and by percentage increase in blood EPA levels post-consumption. In this study, only the former was possible, as EPA levels were highly reliant on the level of EPA supplementation. In a study with equivalent doses of EPA, percentage change in EPA post-supplementation could be utilised to further verify compliance. Furthermore, although compliance by pill count was excellent, there is no way to verify if subjects did indeed fully comply with the protocol, and some of the results may have been a result of lack of compliance, rather than the relative efficacy of the supplement *per se.*

A statistical assessment of the order of treatment showed no effect of treatment order on the results. Likewise, there were no statistical differences among baseline levels of EPA and DHA after each washout period. Nonetheless, it would be useful, in future studies, to measure other biomarkers in addition to levels of omega-3 fatty acids, for example levels of carrier proteins, receptors, etc., in order to ensure that absence of any carry-over effect after each washout period. In addition, diet records and/or food frequency questionnaires during washout periods would be useful in determining both background diet and omega-3 intake of subjects. Alternatively, subjects might be given a list of foods (e.g. fish and fish products) and supplements that would be forbidden from consumption during the washout periods, as this would facilitate equality of baseline levels of omega-3 fatty acids at the beginning of each test period.

## Conclusion

Current research indicates several different roles for the individual ω-3 FAs in the body [39,40], such that the relative improvement in whole blood ω-3 FA levels from supplementation, and the benefits that ensue from that supplementation, may depend upon the purpose of the supplementation for each individual, i.e. patients with a particular condition or disease vs. healthy individuals. Researchers have reported that relative amounts of EPA and DHA in a supplement differentially confer beneficial effects [41-43]. It may be that a small increase in a given ω-3 FA may be sufficient for some individuals, whereas a larger increase may be necessary for patients with specific conditions. This study has shown that for segments of the population hoping to reduce their risk of developing elements of CVD, a judicious decision regarding the type and daily dose of ω-3 supplement will be important. It is clear that not all ω-3 supplements are created equal, at least in terms of CVD risk reduction.

## Subjects and methods

### Subjects

Beginning in May of 2012, a total of 35 healthy participants, 17 females and 18 males, were recruited for the trial via newspaper and social media advertisements in Guelph, Ontario, Canada. The mean age of the group was 34.5 years, with a range of 19 to 60 years. Major inclusion criteria was good health, and exclusion criteria included having taken omega-3 supplements in the previous 3 months, consuming fish on a regular basis (more than one serving per week), any fish or seafood allergy, the diagnosis of any medical illness or conditions, and any gastro-intestinal insufficiency. This study was conducted in accordance with the current version of GCP as defined in the International Conference of Harmonization. Written informed consent was obtained prior to participation in the study, and the informed consent form (ICF) followed the principles defined by the FDA in US 21 CFR part 50 and the ICH Guidelines. The ICF, study protocol and any amendments were approved by an independent Research Ethics Board prior to the commencement of the study. The last patient, last visit was in June of 2013.

### Study design

This was a single centre, open-label, randomized, cross-over comparator study. Eligible participants were randomly assigned to consume one of four products for a period of 4 weeks, followed by a 4-week washout period, and this schedule of four weeks on, four weeks off continued until all participants had consumed each product for a 4-week period. The randomization of the order of treatment substances was generated using an Internet program (http://www.randomization.com) and the procedure was verified using SAS© statistical software at the University of Guelph. Subjects were requested to consume the dose of each supplement that was prescribed on the bottle label. The ω-3 FA content of the four supplements is presented in Table 8.

**Table 8:** Per capsule and total daily dosage composition of four comparator products (CP)

**TRT**	**Product**	EPA & DHA per capsule*	**Tested values**	**Label use: caps/day**	**Daily dosage of EPA + DHA**
**rTG**	Nordic Naturals ProOmega^®^	325 mg EPA	329.6 mg EPA	2	EPA: 650 mg
	Triglyceride	225 mg DHA	226.0 mg DHA		DHA: 450 mg
**EE**	Minami MorEPA^®^	756 mg EPA	774.2 mg EPA	1	EPA: 756 mg
	Platinum Ethyl Ester	228 mg DHA	233.7 mg DHA		DHA: 228 mg
**PL**	Source Naturals ArcticPure^®^	75 mg EPA	78.0 mg EPA	2	EPA: 150 mg
	Krill Oil Phospholipid	45 mg DHA.	46.7 mg DHA.		DHA: 90 mg
**TG**	New Chapter				
	Wholemega^®^ Salmon	90 mg EPA	96.4 mg EPA	2	EPA: 180 mg
	Oil Triglyceride	110 mg DHA	109.5 mg DHA		DHA: 220 mg

*As listed on manufacturer’s label.

Venipuncture blood samples were collected into K-EDTA-anticoagulated tubes at the beginning and end of each 4-week period, and analysed for whole blood fatty acids. Height, weight and blood pressure were measured at each time-point, Body Mass Index (BMI) was calculated, and protocol compliance was measured by capsule count at the end of each 4-week consumption period. The primary outcome measures were change in whole blood levels of EPA, DHA, and EPA + DHA from Day 0 to Day 28. Secondary outcome measures were change in docosapentaenoic acid (DPA) level and in Omega-3:Omega-6 Ratio from Day 0 to Day 28.

### Whole blood fatty acid analysis

The methodology involved quantification of fatty acids by gas chromatography flame ionization detection (GC-FID). Whole blood fatty acids were methylated with acidified methanol, followed by separation of the analytes and delivery of the sample to the flame ionization detector for ionization and detection by the GC system. Fatty acid methyl esters (FAMEs) were separated on a capillary column based on their boiling points. The effluent from the GC column passed through an FID flame (FID consists of a hydrogen/air flame and a collector plate), which broke down organic molecules and produces ions. The ions were collected on a biased electrode and produced an electrical signal. The resulting current was amplified to yield the output signal, which was observed as a peak in the chromatogram. The FID is extremely sensitive with a large dynamic range. Quantification of fatty acid methyl esters was based on one-point calibration curves that were generated for all 25 analytes. Concentration of each fatty acid in an unknown sample was converted into mass by multiplying the concentration (μM) with the molecular weight (g/mol) and volume of the sample (injection volume, 1 μl). The sum of all analytes (g) in a sample was then used to establish the fraction of FAMEs as% weight.

### Statistics

A power analysis indicated that 30 subjects would be required to detect a difference of 0.24% by weight of total EPA, assuming a variance error of 0.33% by weight, a p-value of 0.05 and power of 80%. SAS 9.3 was used for statistical analysis (SAS Institute Inc., Cary, NC). ANOVA for repeated measures (PROC MIXED) was the statistical method used to determine if there were any significant differences from Day 0 to Day 28, for each of the four supplements. To determine if the change from Day 0 to Day 28 was statistically different among the four supplements, PROC LSMEANS, with Tukey’s adjustment for multiple comparisons was employed. All data were also checked for normality, and for homogeneity of variance using Shapiro-Wilks and Brown-Forsyth testing, respectively. All of the directly-measured biomarker results were normally-distributed, as were all of the calculated biomarkers, except for the EPA + DHA percentage change. However, there was homogeneity of variance for both the directly-measured and the calculated biomarkers, including EPA + DHA percentage change; thus, parametric statistical methods were considered appropriate.

## Abbreviations

AA: Arachidonic Acid; BMI: Body Mass Index; BP-D: Blood Pressure - Diagnostic; BP-S: Blood Pressure-Systolic; CRF: Case Report Form; RTG: Comparator Product A- Nordic Naturals^®^ ProOmega^®^; EE: Comparator Product B – Minami MorEPA^®^ Platinum; PL: Comparator Product C – Source Naturals ArcticPure^®^; TG: Comparator Product D – New Chapter Wholemega-3^®^; DHA: Docosahexaenoic Acid; DPA: Docosapentaenoic Acid; EE: Ethyl Ester; EPA: Eicosapentaenoic Acid; FAMEs: Fatty Acid Methyl Esters; FA(s): Fatty Acid(s); FDA: Food and Drug Administration; GC-FID: Gas Chromatograph- Flame Ionisation Detection; HT: Height; ICH: International Conference on Harmonization; μmol: Micromole; Mg: Milligram; NDI: Nutrasource Diagnostics Incorporated; ω-3 FA: Omega-3 Fatty Acids; PL: Phospholipid; SAS: Statistical Analysis System; SD: Standard Deviation; SOP: Standard Operating Procedure; TG: Triglycerides (Triacylglycerol); WT: Weight; ω-3:ω-6: Omega-3:Omega-6 Ratio; ω-6:ω-3: Omega-6:Omega-3 Ratio.

## Competing interests

In the past five years none of the co-authors has received reimbursements, fees, funding, or salary from an organization that may in any way gain or lose financially from the publication of this manuscript, either now or in the future. All three co-authors are employed by Nutrasource Diagnostics Inc., a Contract Research Organisation based in Guelph, Ontario, Canada. Nordic Naturals contracted NDI to design and implement this clinical trial, and they funded the entire trial, including provision of all four of the comparator products tested in this trial, and including any publication costs. NDI has no financial relationship with Nordic Naturals, other than as a third-party CRO being contracted to perform a clinical trial at arm’s length from the sponsor, and being paid for this work. NDI owns the trademark of the OmegaScore™ test. The OmegaScore™ was utilized in this trial as only a diagnostic test for cardiovascular risk from omega-3 fatty acid levels. The study was not designed in any way to support or market the use of the OmegaScore™.

None of the co-authors hold any stocks or shares in an organization that may in any way gain or lose financially from the publication of this manuscript, either now or in the future.

None of the co-authors hold or are currently applying for any patents relating to the content of the manuscript. They have not received reimbursements, fees, funding, or salary from an organization that holds or has applied for patents relating to the content of the manuscript.

None of the co-authors have any other financial competing interests.

There are no non-financial competing interests (political, personal, religious, ideological, academic, intellectual, commercial or any other) to declare in relation to this manuscript.

## Authors’ contributions

ML co-designed the trial, collated the results, completed a final report and wrote this manuscript. CC was the key researcher in the operation and completion of the clinical trial. She enrolled participants, assigned them to interventions, and followed them throughout the trial. WR was co-designer of the trial. A staff person from NDI who was not involved in the clinical trial generated the randomization sequence. All authors read and approved the final manuscript.
